# Demands and Needs for Psycho-Oncological eHealth Interventions in Women With Cancer: Cross-Sectional Study

**DOI:** 10.2196/cancer.7973

**Published:** 2017-11-24

**Authors:** Johanna Ringwald, Lennart Marwedel, Florian Junne, Katrin Ziser, Norbert Schäffeler, Lena Gerstner, Markus Wallwiener, Sara Yvonne Brucker, Martin Hautzinger, Stephan Zipfel, Martin Teufel

**Affiliations:** ^1^ Department of Psychosomatic Medicine and Psychotherapy University Hospital Tuebingen Tuebingen Germany; ^2^ Comprehensive Cancer Center Tuebingen-Stuttgart University Hospital Tuebingen Tuebingen Germany; ^3^ Department of Obstetrics and Gynecology University Hospital Heidelberg Heidelberg Germany; ^4^ Department of Obstetrics and Gynecology University Hospital Tuebingen Tuebingen Germany; ^5^ Department of Psychology Clinical Psychology and Psychotherapy University of Tuebingen Tuebingen Germany; ^6^ Department of Psychosomatic Medicine and Psychotherapy LVR-Clinic Essen University of Duisburg-Essen Essen Germany

**Keywords:** cancer, oncology, eHealth, Internet, needs, Web-based interventions

## Abstract

**Background:**

Over the last decade, a growing body of studies regarding the application of eHealth and various digital interventions has been published and are widely used in the psycho-oncological care. However, the effectiveness of eHealth applications in psycho-oncological care is still questioned due to missing considerations regarding evidence-based studies on the demands and needs in cancer-affected patients.

**Objective:**

This cross-sectional study aimed to explore the cancer-affected women’s needs and wishes for psycho-oncological content topics in eHealth applications and whether women with cancer differ in their content topics and eHealth preferences regarding their experienced psychological burden.

**Methods:**

Patients were recruited via an electronic online survey through social media, special patient Internet platforms, and patient networks (both inpatients and outpatients, University Hospital Tuebingen, Germany). Participant demographics, preferences for eHealth and psycho-oncological content topics, and their experienced psychological burden of distress, quality of life, and need for psychosocial support were evaluated.

**Results:**

Of the 1172 patients who responded, 716 were included in the study. The highest preference for psycho-oncological content topics reached anxiety, ability to cope, quality of life, depressive feelings, and adjustment toward a new life situation. eHealth applications such as Web-based applications, websites, blogs, info email, and consultation hotline were considered to be suitable to convey these content topics. Psychological burden did not influence the preference rates according to psycho-oncological content and eHealth applications.

**Conclusions:**

Psycho-oncological eHealth applications may be very beneficial for women with cancer, especially when they address psycho-oncological content topics like anxiety, ability to cope, depressive feelings, self-esteem, or adjustment to a new life situation. The findings of this study indicate that psycho-oncological eHealth applications are a promising medium to improve the psychosocial care and enhance individual disease management and engagement among women with cancer.

## Introduction

Breast and gynecological cancer and the treatment of these diseases are psychologically challenging for affected women. A variety of physical and psychosocial impairments and lifestyle changes can occur and result in a lower health-related quality of life (QoL) and higher unmet supportive care needs [1-3]. As a consequence, about one-third of women affected by cancer develop high cancer-related distress [4] or clinically relevant syndromes (eg, adjustment disorder, anxiety disorder, and depression) [5]. Up to half of all patients express a need for psycho-oncological care to cope with the disease [6,7]. Previous studies reported that cancer patients with unmet supportive care needs are those who are younger, female, manifest high anxious or depressive scores, live alone [3,8,9], have a lower income [10], or have a lower QoL [11]. Patients with breast, colorectal, blood, lung, and prostate cancer reported higher unmet supportive care needs than patients with melanoma [12]. However, one study showed that colorectal cancer patients expressed lower unmet supportive care needs as compared to breast cancer, lymphoma, and lung cancer patients [11], and a second study demonstrated that breast cancer patients express lower needs than patients with multiple cancer sites, lung cancer, colorectal cancer, brain cancer, and other types of cancer patients [13].

Due to the high cancer-related psychological burden, current international and national cancer guidelines recommend early assessment of and support for psychosocial problems, distress, unmet supportive care needs, problems with daily activities, and lifestyle risks [14,15]. Therefore, screening tools are used to measure the level of distress [16,17].

A variety of psycho-oncology interventions have been developed to support cancer-affected patients during and after treatment to reduce unmet supportive care needs [18-20]. However systematic reviews show that a majority do not benefit from those interventions [21], especially in the long-term, and high distress still persists after several years, especially among younger women (younger than 50 years) with breast cancer. This may indicate that contexts of psycho-oncological interventions do not cover content topics that are relevant enough to sustainably engage patients [8,22]. It has been discussed that psycho-oncological interventions must address specific needs and demands of cancer-affected patients to sustainably improve their well-being [21,23]. Interventions have to be tailored according to patient preferences [23]. Additionally, psycho-oncological care has to reflect living conditions (eg, rural area [24,25] or age [23]). Furthermore, it is important to integrate psycho-oncological care into daily life in order to reduce the barriers of psychosocial care [24]. Digital media has revolutionized our lives as well as the health care industry, and it continues to do so. As technology rapidly improves, many individuals with health problems turn to the Internet to seek out relevant health information [26-30] and take part in Internet-based interventions as an active coping strategy [31,32]. eHealth and digital health have the potential to revolutionize patients’ lives, and eHealth applications, electronic services or systems that support processes and communication in medicine and health care, are changing health care delivery with growing compliance on the part of both patients and health care experts [20,33,34]. Cancer patients represent a growing proportion of health information seekers [30,35]. Over the last decade, a growing body of studies regarding the application of eHealth [29,36,37] and different online interventions [38] have been published and are widely used in psycho-oncological care [39]. While online searches for cancer information, eHealth applications, and online interventions in psycho-oncological care are more common, less is known about cancer patients’ real demands for online and offline psycho-oncological interventions [38,40]. Current online psycho-oncological interventions used the contents of Web-based stress management and depression programs without relying on well-conducted studies in big samples of cancer-affected persons assessing the psychological demands and needs of patients. Other interventions deliver standardized health information and patient education tools to increase patient knowledge about cancer. However, the effectiveness of psycho-oncological care is still not solid due to missing consideration about the real demands and needs for psycho-oncological care, especially in eHealth applications among cancer-affected patients [19,21,38]. McAlpine and colleagues [38] concluded in their review that further psycho-oncological eHealth applications (ePOAs) would benefit from an informed approach and objective evidence to justify the creation and implementation of ePOAs for the cancer population. In Germany, the expansion of eHealth is continuously growing and the revenue is expected to show an annual growth rate of 19.1% [41].

The aims of this cross-sectional study were to describe the cancer-affected women’s needs and demands for psycho-oncological content topics for eHealth applications and determine if women with cancer differ in their demands regarding their experienced psychological burden (distress, QoL, and need for psychosocial support).

## Methods

### Study Design and Recruitment

A total of 1172 women with either breast or gynecologic cancer (or both) were assessed in a cross-sectional approach. Patients were recruited to answer an electronic online survey (Questback) through social media, special patient Internet platforms, self-help group leaders, patient networks (eg, Breast Cancer Aid Germany or BRCA Network), and cancer counseling centers. Duplicate entries were avoided by preventing users with the same IP address further access after completion of the survey. Furthermore, consecutive inpatients and outpatients were asked whether they would like to participate in the study (paper-and-pencil questionnaire in the department of gynecology at the University Hospital Tuebingen, Germany). The self-reported paper-and-pencil or self-reported online questionnaires took participants an average of 20 minutes to complete. Eligibility criteria were defined as an adult (age 18 years and older) with breast cancer, gynecologic cancer, or both with sufficient language skills to complete a set of questionnaires. Participation was voluntary and anonymous; no personal identifying information was collected from the patients. The beginning of the questionnaire included the consenting page. Of the 1172 participants assessed, 41 did not meet the eligibility criteria because of another cancer diagnosis. Incomplete datasets (less than 80% response rate) were excluded, resulting in a final dataset of 716 participants, with 581 surveys completed online and 135 surveys completed as paper-and-pencil questionnaires. The local ethics committee of the University Hospital Tuebingen approved the study protocol.

### Measures

#### Demographic and Disease-Related Information

Demographic variables included age, gender, marital status, and number and age of children. Self-reported data on the type of cancer, time since primary diagnosis, and status of disease (primary disease, metastasis, and recurrence) were also collected.

#### Patient Preference Survey

The patient preference survey was self-generated. In total, 25 items considered the patient preference items for an ePOA. Two categories were created with 19 content topics for a psycho-oncological intervention and 6 possible eHealth applications (Web-based application/info home page, chats and blogs, info email, consultation hotline, phone, video conference). Patients ranked their answer on a 3-point Likert scale ranging from 1=not important to 3=very important to the question, “Which content topic is important for a psycho-oncological intervention?” Next, patients ranked their answer on a 3-point Likert scale ranging from 1=not suitable to 3=very suitable to the question, ”Which application is suitable for the mentioned content topics?”

#### Distress Thermometer

The 11-level visual analog scale of the Distress Thermometer is widely used to measure distress and has been validated in diverse oncology applications [42,43]. Patients were instructed to choose a number indicating how much distress they have been feeling over the past week, including today, between 0=no distress and 10=the worst distress imaginable. A cut-off score of ≥5 is recommended as indicative of a high distress level [42]. A score between 0 and 4 was considered as not distressed, between 5 and 7 as distressed, and between 8 and 10 as highly distressed [44].

#### Hornheider Screening Instrument

The Hornheider Screening Instrument (HSI) is a widely used German 7-item screening instrument to identify patients in need of psychosocial support [45]. The short version of the HSI has been shown to be valid and reliable [46]. It asks for physical condition, mental condition, level of information about illness and treatment, psychosocial distress apart from present illness, distress of relatives, the availability of people to talk to about concerns and anxiety, and the ability to relax during the day. The need for an intervention is indicated when the calculated score is >0.30.

#### Quality of Life

The EuroQoL 5-Dimension 3-Level Questionnaire (EQ-5D-3L) has been used in many clinical trials and methodological studies published in the peer-reviewed literature. It is a standardized instrument for describing and evaluating a patient’s general health status and can be used for clinical assessment of QoL [47]. To measure the QoL, the visual analog scale portion of the EQ-5D-3L was used where own health today is rated on a scale from 0=worst imaginable health to 100=best imaginable health. Values >66 were consider as high QoL, between 51 and 65 as middle QoL, and <50 as low Qol. These cutoffs were analyzed with median splitting within our study group.

### Data Analysis

Descriptive statistics, mean and standard deviation, frequencies, percentages, and chi-square statistics for categorical variables were performed using SPSS 21 for Windows (IBM Corp). Statistical analysis was performed to search for a relationship between patient preferences for psycho-oncological eHealth care and psychological burden with distress, QoL, and need for psychosocial support. Data were normally distributed. Chi-square statistics were used to examine the data for associations between the psychological burden and the preference for psycho-oncological content topics and ePOAs. We computed for distress (highly distressed, distressed, not distressed) and QoL (low, middle, high) in a 3×2 distribution table and for HSI (needing psychosocial support vs not needing psychosocial support) in a 2×2 distribution table. For this purpose, the responses of items were dichotomized (preferences: important vs nonimportant; eHealth applications: suitable vs nonsuitable). Missing data only occurred for the patient preference surveys. The overall mean of missing values was estimated as 2.075%. Missing values were considered only if at least 80% of each of the questionnaires had been completed. Using the Little missing completely at random test, it was confirmed that the data were missing randomly. The expectation-maximization algorithm was used to input the missing data [48]. All of the statistical tests were 2-sided, and *P*<.05 was considered statistically significant.

## Results

### Participants

Of the 1172 patients who responded, 716 (61.09%) datasets met the inclusion criteria, showed acceptable quality, and were included in the study. The mean age of participants was 50.2 (SD 10.3) years (range 25-83 years). Nearly 80.4% (576/716) of the patients were primarily diagnosed with cancer, and 12.2% (87/716) of participants were diagnosed with metastasis; 11.0% (79/716) were experiencing a recurrence of the past cancer diagnosis. The frequencies of other disease-related and demographic variables and mean values and standard deviations of the Distress Thermometer, EQ-5D-3L, and HSI questionnaires are presented in Table 1.

### Relevant Psycho-Oncological Content Topics for eHealth Applications

**Figure 1:**
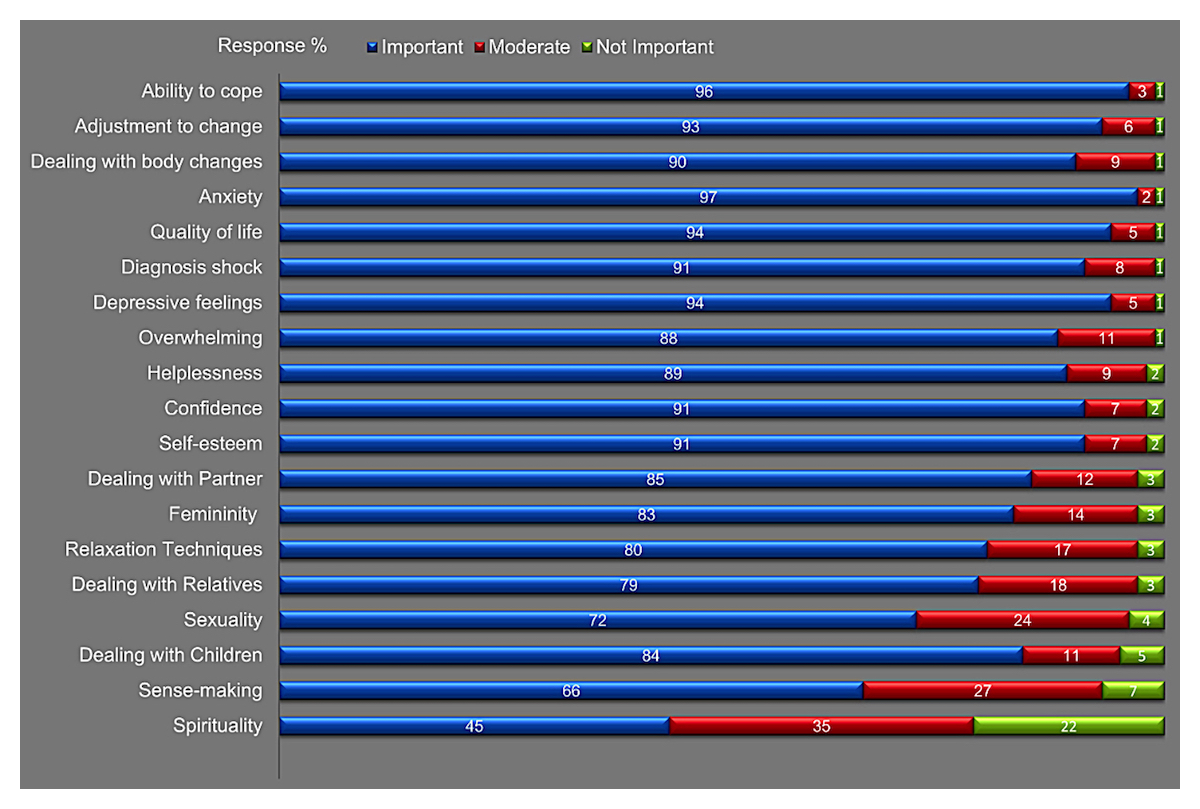
Relevant psycho-oncological content topics for eHealth applications.

The 19 content topics for a psycho-oncological intervention were rated by the patients (see Figure 1). The highest rates reached anxiety (675/697, 96.8%), followed by ability to cope (674/696, 96.8%), QoL (657/696, 94.4%), depressive feelings (655/695, 94.2%), and adjustment to new life situation (654/700, 93.4%).

**Table 1:** Study population characteristics: sociodemographic and disease-related information and psychological burden.

Characteristics		Total
Age, years, mean (SD); range		50.2 (10.3); 25 to 83
Length of time between first diagnosis and questionnaire completion, years, mean (SD); range		4.6 (5.0); 0 to 39
**Cancer diagnosis, n (%)**		
	Breast^a^	652 (91.06)
	Gynecologic^a^	86 (12.01)
**Disease status, n (%)**		
	First episode^a^	576 (80.44)
	Metastasis^a^	87 (12.15)
	Recurrence^a^	79 (11.03)
**Married/with a partner, n (%)**		
	Yes	600 (83.8)
	No	116 (16.2)
**Children, n (%)**		
	0	159 (22.8)
	1	166 (23.2)
	2	241 (33.8)
	3	97 (13.6)
	4	18 (2.5)
	≥5	8 (1.3)
	Data missing	24 (3.4)
**Psychological distress, mean (SD); possible range**		
	Distress Thermometer	5.60 (2.57); 0 to 10
**Quality of life, mean (SD); possible range**		
	EQ-5D-3L	62.77 (19.88); 0 to 100
**Need for psychosocial support, mean (SD); cutoff**		
	Hornheider Screening Instrument	0.66 (1.51); >0.30

^a^Self-reported; multiple answers possible.

Lower preference rates were reached by spirituality (308/692, 44.5%), sense-making (632/692, 66.0%), and sexuality (495/692, 71.5%).

### Patient Preferences Regarding Psycho-Oncological eHealth Applications

**Figure 2:**
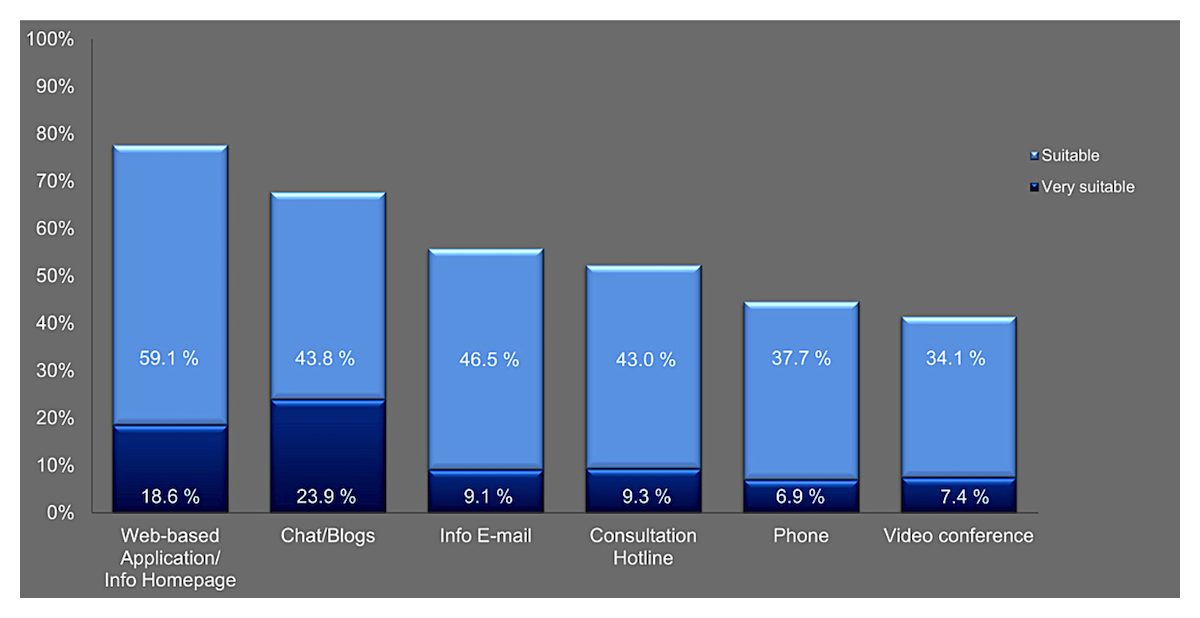
Patient preferences regarding eHealth application to convey psycho-oncological content topics.

Almost all of the eHealth applications for conveying the content topics were considered by more than 50% as suitable. Web-based application/info home page (540/695, 77.7%) was considered to be the most suitable compared to other eHealth applications. Blogs or chats were considered suitable or very suitable (470/694, 67.7%). More than half considered the eHealth applications info email (387/694, 55.8%) and consultation hotline (361/694, 52.0%) suitable or very suitable. Videoconference was judged by the least number of patients (285/687, 41.5%) as suitable for psycho-oncological care (see Figure 2).

### Relationship Between Psycho-Oncological Burden and Perceived Relevance of Content Topics in eHealth Interventions

Preferences for all content topics were equally distributed in the subgroups distress and QoL. Preferences were not dependent on high, middle, or low distress or high, middle, or low QoL. Also, in the context of needing psychosocial support, patients preferred the same content topics for a psycho-oncological intervention. We found no differences between participants with different levels of distress or QoL concerning the preferred content of eHealth interventions. Furthermore, time since diagnosis or prognosis as well as recruitment (eg, hospital vs Facebook) had no influence on needs (data not shown).

## Discussion

### Principal Findings

Our survey explored for the first time the perceived demands and needs for a psycho-oncological eHealth intervention among women with breast and gynecological cancer. Furthermore, we investigated whether there is a relationship between psychological burden (distress, QoL, and need for psychosocial support) and content topic relevance. In this sample of 716 cancer-affected women, we found distinctively relevant content topics for eHealth interventions. The content topics of ability to cope, anxiety, depressive feelings, or adjustment to new life situations have a high impact on eHealth interventions and, in turn, reflect the needs and demands for psycho-oncological care of cancer-affected women. Spirituality, sense-making, and dealing with children had no high relevance for eHealth interventions in our sample. Furthermore, Web-based application/info home page, info email, and chats and blogs were identified as very suitable and suitable for conveying psycho-oncological content topics to the patients. We found that preferences for specific content topics and eHealth applications were equal between patients with high and low burden (experienced distress and QoL). In addition, the need for psychosocial support did not influence the demands and needs of the patients. To summarize, interestingly, women with cancer experience—independently of their psychological burden—have the same demands and needs for psycho-oncological content topics. Furthermore, they expressed the same demands for eHealth applications in psycho-oncological care.

### Interpretation of our Findings

We found in our survey that the most preferred type of psycho-oncological eHealth interventions are Web-based applications or info emails. These results are in line with previous studies that also identified high preferences for eHealth applications [10,33,34,49]. Moreover, we found that eHealth applications may have potential beneficial effects for specific psycho-oncological content topics like anxiety, coping, depressive feelings, self-esteem, or adjustment to new life situations (see Figure 1). It seems that ePOAs have the potential to support cancer-affected women in the context of delivering information, feelings of control, self-efficacy, or self-management during the time of dealing psychologically with the disease. Jansen et al [50] also found an overall positive attitude toward self-management and eHealth among different cancer survivors. Furthermore, they determined that men were more likely to report supportive care needs regarding healthy lifestyle programs, and they are in general highly interested in eHealth. Børøsund et al [51] described the use of patterns in Web-based illness management support among prostate and breast cancer patients. Regarding the use of Web-based support applications, they determined that lower levels of social support and higher depression scores were more influential among women with breast cancer, and symptom distress was more influential for men with prostate cancer. It seems that cancer-affected men are more likely to participate in eHealth applications that offer lifestyle elements, and women with cancer are more willing to participate in eHealth applications that also contain psycho-oncological elements.

The psycho-oncological content topics that were evaluated for eHealth applications (see Figure 2) are similar to the topics discovered in other studies. Different researchers discovered high unmet psychological and psychosocial needs in cancer patients and reported that it is urgently necessary to further evaluate and address these specific demands and needs in future and modern eHealth intervention [9,52,53]. In our survey, sexuality, sense-making, and spirituality were considered to be less important, a finding which diverges from other research findings [54,55]. It could be that these content topics are more suitable for face-to-face interventions and are not suitable for eHealth interventions. The content topic self-esteem was rated extremely high and considered to be very important by the patients for an eHealth intervention. In previous psycho-oncological interventions, this content topic was mostly neglected and not taken into account, especially in eHealth interventions. Furthermore, we have found that more than 50% of the patients reported a high preference for ePOAs independently of the experienced psychological burden. This is in line with the findings of Jansen et al [50] who reported that the perceived needs for supportive care, including healthy lifestyle programs, were highly accepted, and in general, cancer survivors had a positive attitude toward eHealth. Different from our findings, Jansen and colleagues [50] found that the attitude was associated with QoL [50]. An et al [30] demonstrated that cancer patients perceived more social support from the Internet when they actively posted or shared contents than when they used the Internet solely as an informational resource [30]. We also determined that chats and blogs were highly accepted by our patients. In addition, studies reported that future psycho-oncological interventions should consider daily practice and the local accessibility as ePOAs have the potential to close this gap [24,56,57]. Additionally, cancer survivors were positive toward ePOAs that enable them to enhance their own QoL and support them in finding tailored supportive care [57]. It was shown that eHealth applications are well accepted for therapy assistance in general (like patient-physician communication) and eHealth programs as a part of usual health care may be promising [29,50,57]. A promising result of our study is that a substantial group of participants in need of supportive care prefer ePOAs for the delivery of adequate psychosocial care. Furthermore, they rate eHealth as adequate for specific psycho-oncological content topics like anxiety, ability to cope, depressive feelings, self-esteem, or adjustment to new life situations.

### Strengths and Limitations

Our survey study was based on a large sample (N=716) of patients diagnosed with breast cancer, gynecologic cancer, or both. Our use of various and novel recruitment strategies (Internet links, Facebook, blogs, flyers) led to a large proportion being included through the online questionnaire (n=581). This shows that eHealth is especially targeting patients with eHealth literacy, and therefore our results can be considered as representative of these patients [58]. Nevertheless, there are limitations in the sample selection and generalizability of this survey. Our survey cohort was homogenous, mostly younger, white, and highly distressed. Furthermore, it is important to note that mainly women with breast cancer (91.1%) participated in our survey. Therefore, a recruitment bias can be assumed in our study. However, this trend has been observed in similar studies, and it also reflects reality [51]. Breast cancer has the highest tumor prevalence among women, and various studies show that this patient group suffers mostly under high psychological distress [4,5] and younger patients prefer eHealth applications more than older patients. Further studies including other tumor entities and male patients are needed. Our results agree with findings of former studies [50,51]. The lack of diversity also does not allow extrapolation of study results to statements concerning other tumor entities and men. Our self-generated patient preference survey has not undergone formal reliability and validity testing. Hence, a validated questionnaire for these research questions did not exist when the study was performed.

### Conclusion and Clinical Implications

Our findings show high preference rates for eHealth applications independently of experienced psychological burden among women with cancer. Furthermore, ePOAs may have a high benefit for women with cancer, especially when they address psycho-oncological content topics like anxiety, ability to cope, depressive feelings, self-esteem, or adjustment to new life situations. ePOAs have the potential to help patients overcome disease-related burden and reduce barriers in psychosocial care. However, they can encourage patients who they believe are not sufficiently burdened to participate in common psycho-oncological interventions [24,59]. Our findings encourage the development of further innovative ePOAs that specifically focus on the evaluated psycho-oncological content topics (see Figure 1). In summary, the findings of this study indicate that ePOAs are a promising medium to improve psychosocial care and enhance individual disease management among women with cancer.

## Abbreviations

ePOA: psycho-oncological eHealth application
QoL: quality of life
HSI: Hornheider Screening Instrument
